# Habitat characteristics and insecticide susceptibility of *Aedes aegypti* in the Ifakara area, south-eastern Tanzania

**DOI:** 10.1186/s13071-020-3920-y

**Published:** 2020-02-07

**Authors:** Najat F. Kahamba, Alex J. Limwagu, Salum A. Mapua, Betwel J. Msugupakulya, Dickson S. Msaky, Emmanuel W. Kaindoa, Halfan S. Ngowo, Fredros O. Okumu

**Affiliations:** 1 Environmental Health and Ecological Sciences Department, Ifakara Health Institute, P. O. Box 53, Ifakara, Tanzania; 2 Faculty of Health Science, School of Public Health, University of the Witwatersrand, Johannesburg, South Africa; 3 School of Life Science and Biotechnology, Nelson Mandela African Institution of Science and Technology, P. O. Box 447, Arusha, Tanzania; 4 Institute of Biodiversity, Animal Health and Comparative Medicine, University of Glasgow, Glasgow, G128QQ UK

**Keywords:** *Aedes aegypti*, Habitat characteristics, Insecticide susceptibility, Ifakara Health Institute, Dengue, Chikungunya, Tanzania

## Abstract

**Background:**

*Aedes*-borne diseases such as dengue and chikungunya constitute constant threats globally. In Tanzania, these diseases are transmitted by *Aedes aegypti,* which is widely distributed in urban areas, but whose ecology remains poorly understood in small towns and rural settings.

**Methods:**

A survey of *Ae. aegypti* aquatic habitats was conducted in and around Ifakara, a fast-growing town in south-eastern Tanzania. The study area was divided into 200 × 200 m search grids, and habitats containing immature *Aedes* were characterized. Field-collected *Ae. aegypti* were tested for susceptibility to common public health insecticides (deltamethrin, permethrin, bendiocarb and pirimiphos-methyl) in the dry and rainy seasons.

**Results:**

Of 1515 and 1933 aquatic habitats examined in the dry and rainy seasons, 286 and 283 contained *Aedes* immatures, respectively (container index, CI: 18.9–14.6%). In the 2315 and 2832 houses visited in the dry and rainy seasons, 114 and 186 houses had at least one *Aedes*-positive habitat, respectively (house index, HI: 4.9–6.6%). The main habitat types included: (i) used vehicle tires and discarded containers; (ii) flowerpots and clay pots; and (iii) holes made by residents on trunks of coconut trees when harvesting the coconuts. Used tires had highest overall abundance of *Ae. aegypti* immatures, while coconut tree-holes had highest densities per habitat. *Aedes aegypti* adults were susceptible to all tested insecticides in both seasons, except bendiocarb, against which resistance was observed in the rainy season.

**Conclusions:**

To our knowledge, this is the first study on ecology and insecticide susceptibility of *Ae. aegypti* in Ifakara area, and will provide a basis for future studies on its pathogen transmission activities and control. The high infestation levels observed indicate significant risk of *Aedes*-borne diseases, requiring immediate action to prevent potential outbreaks in the area. While used tires, discarded containers and flowerpots are key habitats for *Ae. aegypti*, this study also identified coconut harvesting as an important risk factor, and the associated tree-holes as potential targets for *Aedes* control. Since *Ae. aegypti* mosquitoes in the area are still susceptible to most insecticides, effective control could be achieved by combining environmental management, preferably involving communities, habitat removal and insecticide spraying.
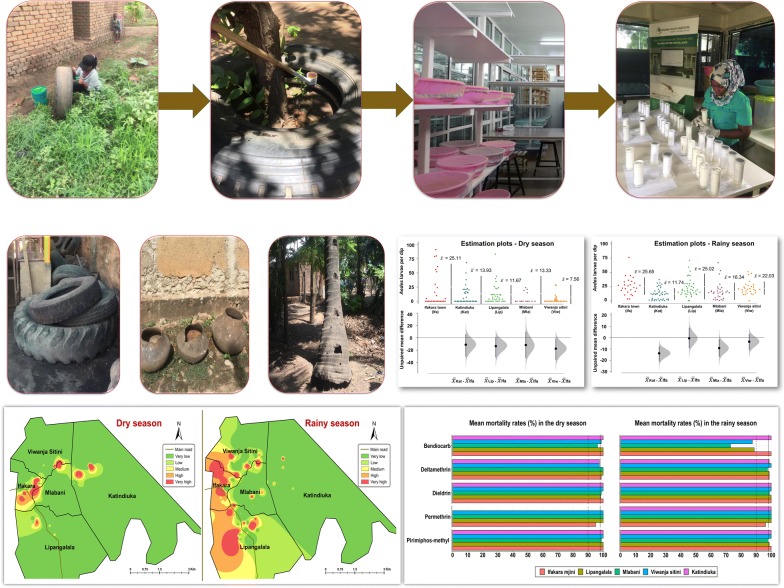

## Background

In recent decades, significant attention has been paid to control mosquitoes that transmit malaria, leading to substantial progress since 2000 [1, 2]. However, other mosquito-borne diseases, such as dengue, yellow fever, chikungunya and Zika, which are transmitted by *Aedes* mosquitoes remain largely neglected. Golding et al. [3] showed that more than 90% of people at risk of vector-borne diseases are affected by at least two such diseases, malaria and dengue fever being the most prevalent. The WHO Global Vector Control Response (GVCR) initiative therefore recommended integrated approaches to address multiple vectors and vector-borne diseases [4]. Unfortunately, unlike malaria, for which effective prevention and treatment options are widely available, the *Aedes*-borne diseases still rely mostly on personal protection measures [5], even though vaccine trials are increasingly advanced [6].

In Tanzania, concerns about *Aedes*-borne diseases have become increasingly prominent in recent years, due to multiple outbreaks, detection of the viruses in humans, and widespread distribution of the *Aedes* mosquitoes [7–10]. Dengue cases have been reported in multiple regions in the country, including Dar es Salaam city, the islands of Zanzibar and Pemba, Mbeya and Iringa areas in the southern Tanzania, as well as Kilimanjaro in the north [11–13]. The most recent outbreak occurred in May 2019, when 1012 new cases were confirmed over just two weeks [14]. By September 2019, 6912 cases had been reported, including 13 deaths [14]. Most outbreaks of *Aedes*-borne diseases have been observed in urban areas, where densities of both the vectors and humans are high [11]. However, human mobility has also led to introduction of the viruses in rural areas and small towns [7]. Unfortunately, efforts against these diseases are hampered by the lack of proper medication or diagnostics [15–17]. Effective vector surveillance and control to prevent potentially infectious mosquito bites therefore remain core components of programmes targeting *Aedes*-borne diseases [5].

Current understanding of *Aedes* mosquitoes is largely based on studies in urban areas where the vector is most widespread [18]. *Aedes aegypti,* the most important of the *Aedes* species, is considered highly anthropophilic, and is a frequent breeder in artificial containers [19], common in urban settings [8]. Improper disposal of waste containers provides perfect breeding environment for *Ae. aegypti* mosquitoes. For example, in coastal Tanzania, used tires and disposed containers were identified as the most common aquatic habitats for *Ae. aegypti* [11, 20]. However, less is known regarding the ecology of these vectors in inland Tanzania, including small towns and rural settings. Yet, this is important to understand distribution of the vectors across the country, and more importantly to prevent introduction or spread of *Aedes*-borne arboviruses. To ensure effective control, such ecological studies should be complemented with investigations on susceptibility to commonly used public health insecticides [21, 22].

This study was therefore conducted to fulfil three key objectives: (i) investigate spatial distribution of *Ae. aegypti* in Ifakara town in south-eastern Tanzania and its surrounding wards; (ii) characterize aquatic habitats of the mosquitoes in the area; and (iii) assess susceptibility of the mosquitoes to insecticides commonly used for vector control.

## Methods

### Study area

**Fig. 1:**
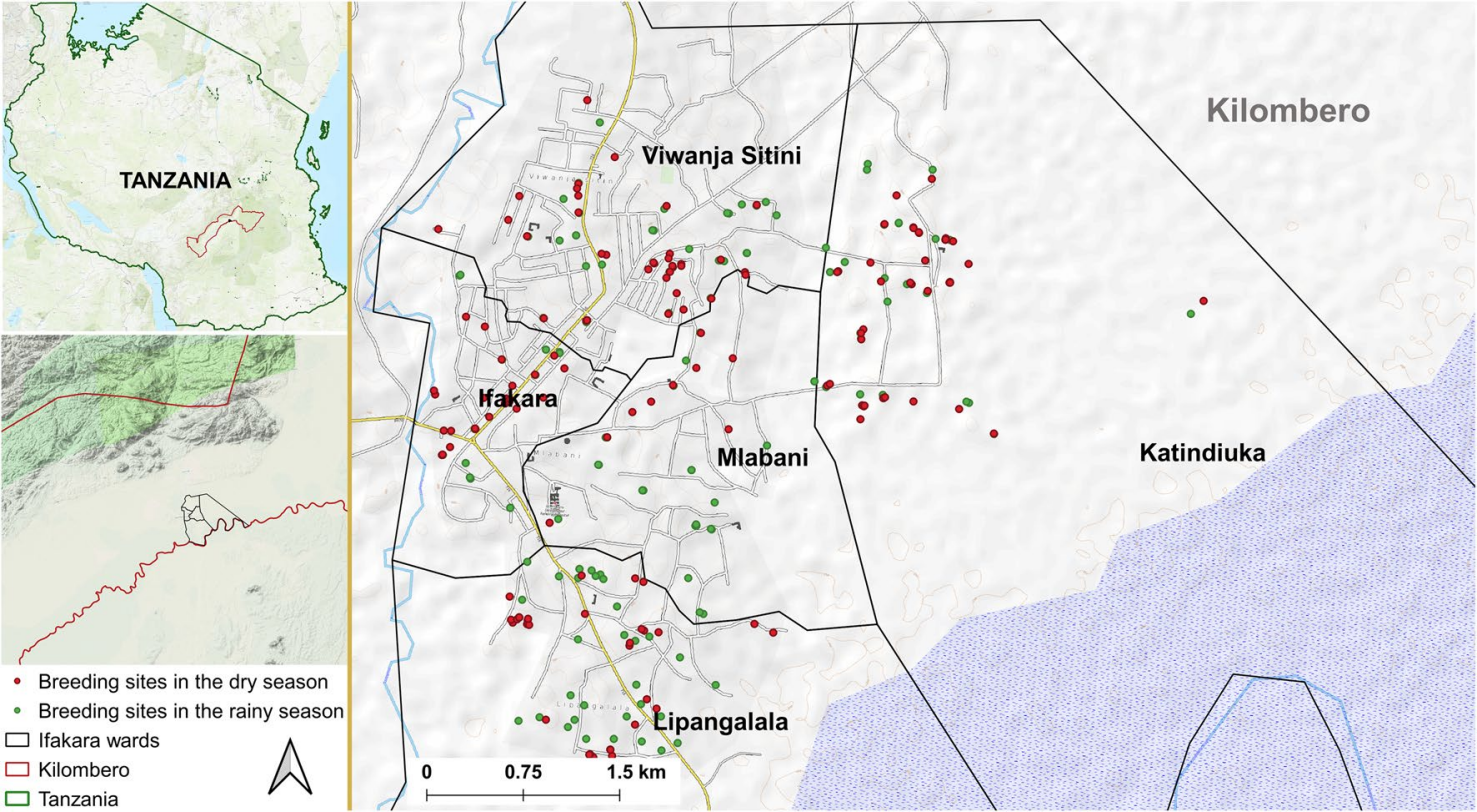
Study area: map of Ifakara town and its surrounding wards showing locations where *Ae. aegypti* immatures were sampled in dry and rainy seasons

Surveys for *Aedes* immatures were conducted in Ifakara town and surrounding wards, namely, Lipangalala (− 8.16428, 36.68964), Viwanja Sitini (− 8.13512, 36.68413), Mlabani (− 8.13952, 36.68964) and Katindiuka (− 8.13154, 36.71165), all in the Kilombero valley in south-eastern Tanzania (Fig. 1). Ifakara town and Viwanja Sitini are characterized as urban, while the other three locations are rural. The area has an average altitude of 270 m, annual rainfall of 1200–1800 mm, relative humidity of 51–71%, and daily temperatures of 20–32.6 °C [23]. The area experiences short rains in November and December, which is interrupted by dry months from January to March. Heavy rains continue from April to May or June, followed by dry July and September. This area is growing rapidly with total population now estimated at 67,500, based on the 2.7% annual growth from the last census in 2012 [24].

### Selection of sampling sites

**Fig. 2:**
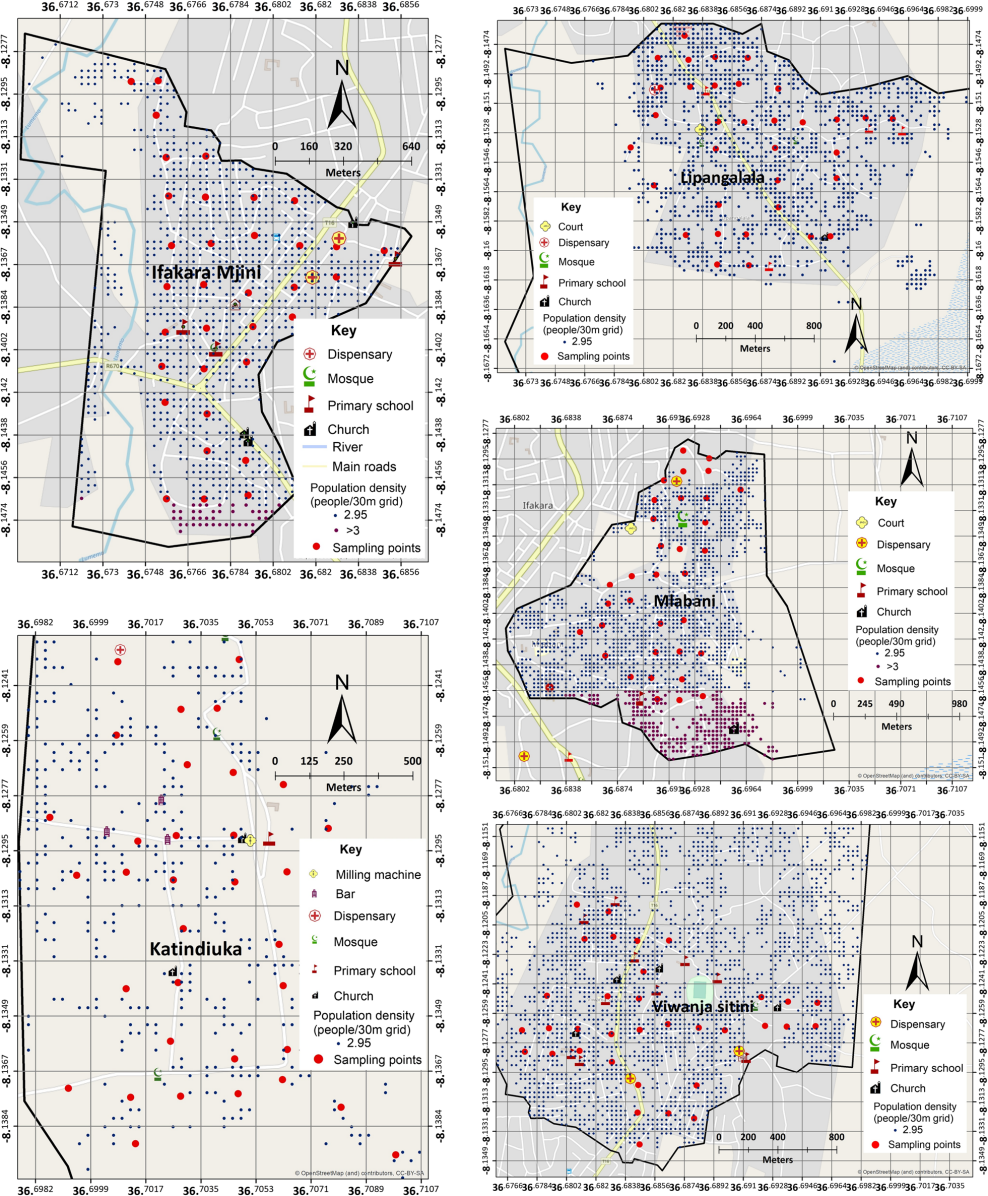
Selected grids in the study area, which were sampled for conducting *Ae. aegypti* larval surveys in dry and rainy seasons. Estimated population densities are also shown

The study area was divided into grids measuring 200 × 200 m, in ArcGIS 10.4 environment (ESRI, USA) as previously described by Mwangungulu et al. [25], and the grids assigned unique identifiers (Fig. 2). The grids were overlaid with household geolocation data initially collected by Ifakara Health Institute’s Health and Demographic Surveillance System [26]. The data were updated using population density maps from Google satellite imagery and a high-resolution settlement layer (HRSL), created by the Facebook Connectivity Lab and Centre for International Earth Science Information Network (CIESIN) [27].

From each ward, 34 grids containing human habitation or other actively used buildings were selected as search grids. For each search grid, houses or buildings nearest to the centroid were identified as starting points for *Aedes* habitat searches. Where no informed consent was obtained, the next nearest consenting household was selected. From the starting points, all potential aquatic *Aedes* habitats were searched within 100 m radii, visiting each search grid twice in the dry season and twice in the rainy season. In total, 170 search grids were visited in each round of the survey. Important features such as schools, marketplaces, worship areas, health facilities and water pumps were also mapped using handheld GPS receivers (Magelan eXplorist GC, USA).

### Sampling of mosquito immatures and characterization of their aquatic habitats

Sampling for *Aedes* immatures and characterization of their habitats was focused on natural and artificial water-holding objects such as tree-holes, used tires, wells and discarded containers and animal feeding containers. Others included coconut shells, tarpaulins, broken grasses and other small objects that could potentially hold water longer than three days. All sites with *Ae. aegypti* larvae or pupae were georeferenced using handheld GPS. The habitats were characterized by: (i) location; (ii) size; (iii) apparent water color; (iv) presence of vegetation; (v) presence of shading; (vi) source of water in the habitat; (vii) whether the habitat was movable or not; and (viii) environmental and social activities surrounding the habitats.

Larvae and pupae were sampled from each of the identified habitats using standard 350 ml dippers, or a smaller 70 ml dipper in cases where habitats were too small to sample using the standard dipper. The larvae and pupae were placed in white trays for morphological identification, using pictorial keys created by the USA Centers for Disease Control and Prevention [28]. They were then sorted, counted and data recorded by habitat type, location and survey instance. The *Aedes* mosquito sampling was done from November 2018 to May 2019, with a break in February 2019.

### Mosquito rearing and identification of emergent adults

The sampled *Aedes* larvae and pupae from the different habitats were transferred to the vector biology laboratory (VectorSphere) at Ifakara Health Institute (IHI) for rearing and eventual morphological identification of emergent adults. The larvae were fed on Tetramin® baby fish food and maintained at temperatures of 26 ± 2 °C and a relative humidity of 82 ± 10%. Pupae were collected each morning, counted and transferred to netting cages (30 × 30 × 30 cm). Emergent adults were identified under stereomicroscopes using taxonomic keys for *Aedes* mosquitoes [28].

### Bioassays for insecticide susceptibility tests

Bioassays were performed following WHO insecticide susceptibility test guidelines [29, 30]. Female *Ae. aegypti*, 3–5 days-old, originating from each ward were tested against public health insecticides as follows: two pyrethroids (deltamethrin: 0.05%; and permethrin: 0.75%), one organochloride (dieldrin: 4%), one organophosphate (pirimiphos-methyl: 0.25%) and one carbamate (bendiocarb: 0.1%). The two pyrethroids included both types I & II, respectively. The insecticides were selected because they represented the common classes of insecticides for public health generally and in the study area. In each experiment 120–150 mosquitoes were used, so that there were 20–25 individuals per test. Untreated controls were included, and the mosquitoes were initially observed for 60 minutes to observe knockdown at 10, 15, 20, 30, 40, 50 and 60 min. The exposed and non-exposed mosquitoes were then provided with 10% glucose and maintained at 28.0 ± 1.0 °C and 80 ± 10% relative humidity, then overall mortality observed after 24 hours.

### Measurements of mosquito wing lengths

Emergent adults from different wards were assessed by measuring their wing lengths. All the mosquitoes had been originally collected as larvae or pupae. The mosquitoes were anaesthetized at − 10 °C for 5 min. Wings were then removed from male and female mosquitoes (either the left or the right wing). Drops of distilled water were used to fix the wings onto glass slides. Wing lengths were measured, as the distance from the apical notch to the auxiliary margins, under stereo zoom microscope at 10× magnification using a micrometer ruler.

### Data analysis

Statistical analyses were carried out in the open-source R statistical software, version 3.231 [31]. Descriptive analysis was performed to compare larval densities in different wards and seasons. Densities obtained from the 70 ml dipper were compared to those from the standard 350 ml dipper and a correlation coefficient calculated across all collections. Using this coefficient, the densities assessed by the small dipper were all converted into the standard dipper, so that all subsequent analyses were done on the standard dipper.

Generalized linear models with Poisson distributions for count data were used to model the number of larvae collected per dipper as a response variable against season and habitat type as a fixed factor. Logistic regression was also used to assess associations between positivity of different habitat types for *Aedes* larvae (proportion of individual habitats of each type that had *Aedes* larvae or pupae). The relative risk (RR), odds ratios (OR) and their 95% confidence intervals (95% CI) were estimated. The *dabestr* package was used to assess mean differences in larval abundance between wards and seasons.

Larval indices, namely container index (CI, the proportion of containers infested with *Ae. aegypti* larvae or pupae), house index (the proportion of houses infested with *Ae. aegypti* larvae or pupae) and Breteaux Index (the number of infested containers per 100 houses) were also calculated by ward or season [22, 32, 37].

Mosquito wing lengths were compared using one-way ANOVA, followed by Tukey’s *post-hoc* tests to assess mean differences between wards for both male and female mosquitoes. Susceptibility status of *Ae. aegypti* was computed according to the WHO guidelines [29] and log-probit analysis was used to compute mean duration at which 50% (KD_50_) and 95% (KD_95_) of the exposed mosquitoes were knocked down.

Spatial and seasonal distribution of *Aedes* immatures were analyzed by geostatistical in ArcGIS 10.4 (ESRI, USA). Inverse distance weighted (IDW) interpolation technique [33, 34] was used to visualize the areas with high larval densities. Representation of IDW maps show patterns based on the distance from one observed point to another. Known values (number of larvae) were used as key input feature to estimate unknown locations within 400 m range based on estimated average flight range of *Aedes* mosquitoes [35, 36, 37]. Geoprocessing extents and masks were defined to match the study area.

## Results

### Larval indices

A total of 1515 breeding sites were visited in the dry season and 1933 in the rainy season. Of these, 286 (18.87%) in the dry season and 283 (14.64%) in the rainy season were positive for *Aedes* immatures. The proportions of *Aedes*-infested habitats and houses varied across wards and seasons (Table 1). In the dry season, high container indices (CIs) were observed in Katindiuka, Viwanja Sitini and Ifakara town wards, while in the rainy season, high CIs were recorded in Ifakara town, Viwanja sitini and Lipangalala wards.

**Table 1:** Summary of *Ae. aegypti* larval survey indices by ward and season

Wards	Dry season			Rainy season		
	CI (%)	HI (%)	BI (%)	CI (%)	HI (%)	BI (%)
Ifakara town	21.4	4.18	16.74	27.4	7.12	9.54
Katindiuka	18.7	4.45	9.37	11.2	6.78	7.22
Viwanja sitini	29.5	6.67	20.28	26.2	6.75	15.25
Mlabani	13.0	3.33	5.12	11.9	6.58	10.53
Lipangalala	21.4	6.44	8.44	19.6	5.11	11.11

*Abbreviations*: CI, container index (ratio of larval infested to total inspected containers); HI, house index (ratio of larval infested to all inspected houses); BI, Breteau index (ratio of positive containers per 100 houses inspected)

With regard to house indices (HI), 2315 and 2832 houses were visited in the dry and rainy season surveys, of which, 114 (4.9%) and 186 (6.6%) had at least one positive habitat, respectively. Viwanja sitini ward had the highest HI during the dry season, while Ifakara town had the highest HI in the rainy season. Compared to the dry season, HI increased during the rainy season in all wards expect Lipangalala (Table 1). It was also observed that Viwanja Sitini ward had highest Breteaux Index (BI) in both seasons.

### Densities of *Ae. aegypti* immatures, their distribution and aquatic habitats

A total of 63,470 larvae or pupae were collected from all wards. Of these, 76.3% (*n* = 48,459) were *Ae. aegypti*, 20.9% (*n* = 13,253) were *Culex*, and 2.8% (*n* = 1758) were identified as other *Aedes* spp. In the dry season surveys, Ifakara town produced nearly one third of all immature *Aedes* and more than one third of immature *Culex*. In the rainy season however, Viwanja Sitini had more than one third of the *Aedes* immatures, while Katindiuka produced more than half of all *Culex*. Most *Culex* were found in the dry season, while *Aedes* were more prevalent in the rainy season (Table 2).

**Table 2:** Sampled populations of *Aedes* and *Culex* larvae collected in all aquatic habitats in Ifakara town and its surrounding wards

Wards	Dry season				Rainy season				Total	
	*Aedes*		*Culex*		*Aedes*		*Culex* larvae		*Aedes*	*Culex*
	*n*	%	*n*	%	*n*	%	*n*	%	*n*	*n*
Ifakara town	5325	32	4217	39	6769	20	0	0	12094	4217
Katindiuka	2845	17	1240	11	2383	7	919	37	5228	2159
Viwanja sitini	3527	21	3116	29	11652	35	0	0	15179	3116
Mlabani	1833	11	826	8	7698	23	15	1	9531	841
Lipangalala	3284	20	1386	13	4901	15	1534	62	8185	2920

*Note*: n denotes number of larvae collected, % denotes percentage of larvae by ward

**Fig. 3:**
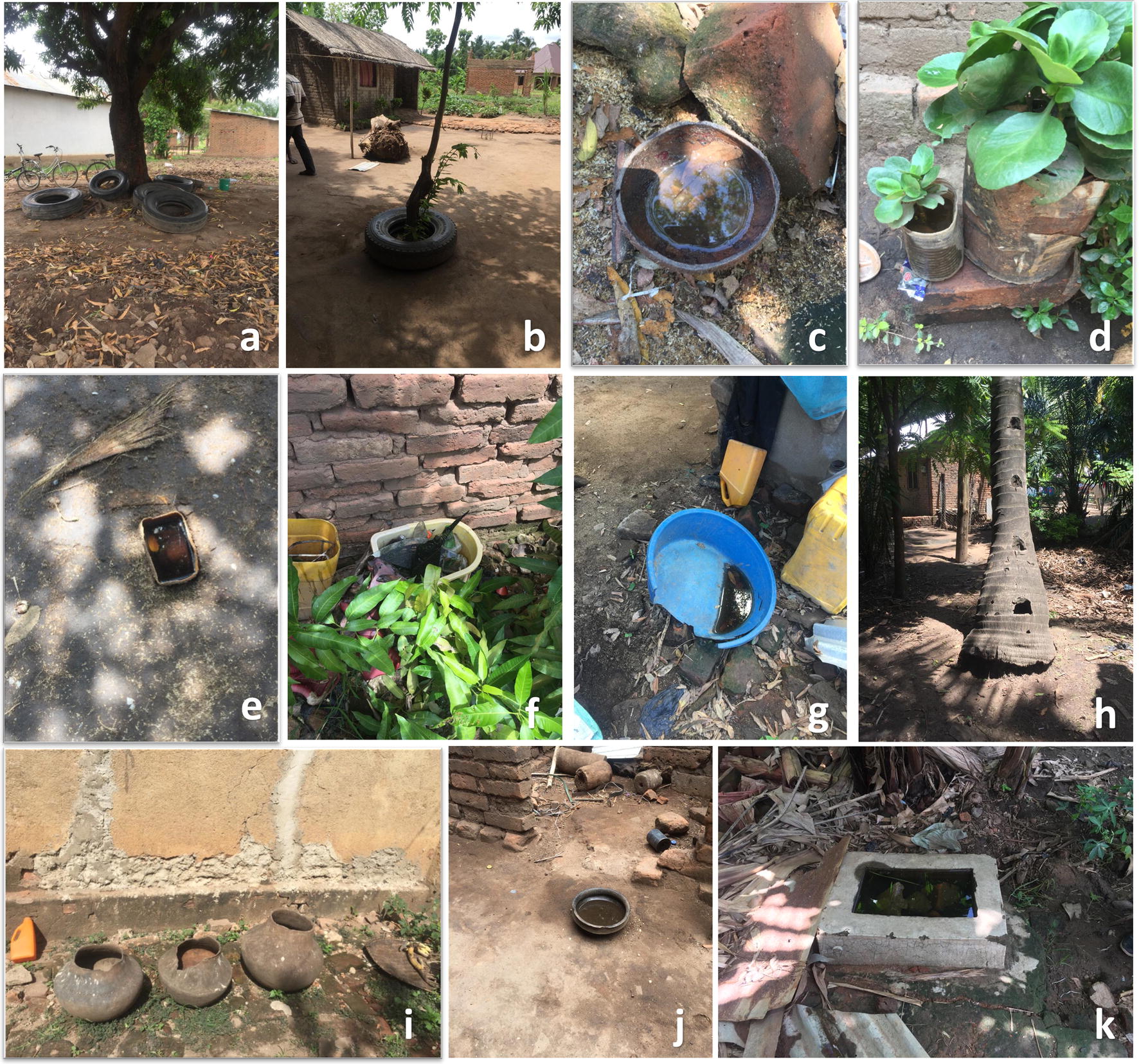
Various breeding sites identified in the study area: **a** used vehicle tires, here repurposed by residents as seats; **b** used tires kept for protecting trees from pests; **c** disposed coconut shells; **d** flowerpots; **e** animal feeding container; **f** broken grasses; **g** disposed containers; **h** coconut tree-holes; **i** clay pots; **j** small containers; and **j** pits such as those at construction sites, in garages, or inspection chambers in waterworks

Overall, most *Aedes* larvae were obtained from used tires and clay pots followed by other containers such as discarded tins, buckets, drums and animal feeding pots (Fig. 3). However, coconut tree-holes and flowerpots had far higher numbers of larvae per dip compared to all other habitat types, in the dry season (Table 3). Likelihood of getting larvae in individual tree-holes was three times higher than in used vehicle tires (RR: 3.00, 95% CI: 2.58–3.50, *P* < 0.01). However, in the rainy season, higher larval densities were observed in other habitats (Table 3).

**Table 3:** Larval densities in different aquatic habitats of *Ae. aegypti* mosquitoes in the dry and rainy seasons in the study area

Habitat type	No. of larvae	No. of habitats	Mean (95% CI)	RR (95% CI)	*P*-value
Dry season					
Used tire	844	51	16.5 (15.46–17.70)	1	
Clay pot	652	44	14.8 (13.7–16.00)	0.89 (0.80–0.99)	0.034
Container	93	24	3.9 (3.16–4.75)	0.23 (0.19–0.29)	< 0.01
Flowerpot	163	9	18.1 (15.53–21.12)	1.09 (0.93–1.29)	0.292
Pit	96	7	13.7 (11.23–16.75)	0.83 (0.67–1.02)	0.081
Tree-hole	199	4	49.8 (43.3–57.17)	3.00 (2.58–3.50)	< 0.01
Others	12	6	2.0 (1.14–3.52)	0.12 (0.07–0.21)	< 0.01
Rainy season					
Used tire	1276	55	23.2 (21.96–24.51)	1	
Clay pot	978	55	17.8 (16.7–18.93)	0.77 (0.70–0.83)	< 0.01
Container	504	27	18.7 (17.11–20.37)	0.80 (0.72–0.89)	< 0.01
Flowerpot	273	17	16.1 (14.26–18.01)	0.69 (0.61–0.79)	< 0.01
Pit	133	7	19.0 (16.03–22.52)	0.82 (0.69–0.98)	0.028
Tree-hole	68	4	17.0 (13.4–21.56)	0.73 (0.57–0.94)	0.012
Others	119	5	23.8 (19.87–28.48)	1.03 (0.85–1.24)	0.790

*Notes*: Category used as reference R = 1, means reported here are predicted from generalized linear model which is average of larvae per dipper to number of breeding sites. Used tire was selected as reference because they were present in all study sites. “Others” included positive breeding sites such as disposed shoes, coconut shells, tarpaulins, broken glasses and open plastic bottles

*Abbreviations*: RR, risk ratio; CI, confidence interval

### Positivity of different habitat types for *Ae. aegypti* immatures

Positivity of the habitats for *Ae. aegypti* are summarized in Table 4. By assessing proportions for each type of habitat, it was determined that used tires were the most commonly infested with *Ae. aegypti* (89% positivity), followed by containers (86% positivity) and clay pots (82% positivity), garage pits (64% positivity) and others (90% positivity). Majority of the positive breeding sites were movable, associated with human activities, or were found in and around residential areas, commercial places and garages. Significantly higher *Ae. aegypti* positivity was observed in the rainy season than in the dry season. Additionally, the number of positive habitats was higher if they had clear water than turbid water.

**Table 4:** Results of the logistic regression analysis showing positivity and negativity of habitats of different characteristics for immature *Ae. aegypti* mosquitoes

Parameter	Category	Positive	Negative	Total	Univariate		Multivariate	
		*n* (%)	*n* (%)		OR (95% CI)	*P*-value	OR (95% CI)	*P*-value
Habitat type	Used tires	89 (84)	17 (16)	106	1		1	
	Clay pot	81 (82)	18 (18)	99	0.86 (0.42–1.78)	0.68	0.55 (0.21–1.42)	0.216
	Container	44 (86)	7 (14)	51	1.20 (0.46–3.11)	0.70	1.07 (0.33–3.48)	0.904
	Flowerpot	22 (85)	4 (15)	26	1.05 (0.32–3.44)	0.93	0.62 (0.13–2.95)	0.551
	Pits	9 (64)	5 (36)	14	0.34 (0.10–1.15)	0.08	0.11 (0.01–2.54)	0.172
	Tree-hole	7 (88)	1 (12)	8	1.34 (0.15–11.58)	0.79	0.92 (0.03–31.86)	0.962
	Others	10 (90)	1 (9)	11	1.91 (0.23–15.91)	0.55	2.98 (0.26–34.82)	0.383
Size	Large	36 (71)	15 (29)	51	1		1	
	Medium	129 (84)	25 (16)	154	2.15 (1.03–4.50)	0.042	1.73 (0.71–4.18)	0.2165
	Small	97 (88)	13 (12)	110	3.10 (1.35–7.17)	< 0.001	0.98 (0.33–2.89)	0.966
Season	Dry season	97 (67)	48 (33)	145	1		1	
	Rainy season	165 (97)	5 (3)	170	16.30 (6.30–42.40)	< 0.001	19.73 (6.61–58.94)	< 0.001
Movability	Immovable	20 (74)	7 (26)	27	1		1	
	Movable	242 (84)	46 (16)	288	1.80 (0.74–4.60)	0.192	0.36 (0.03–5.24)	0.46
Turbidity	Clear	145 (88)	20 (12)	165	1		1	
	Turbid	109 (83)	22 (17)	131	0.68 (0.36–1.32)	0.254	0.79 (0.38–1.67)	0.5417
	Very turbid	8 (42)	11 (58)	19	0.10 (0.03–0.27)	< 0.001	0.13 (0.04–0.44)	< 0.001
Shades	Full	115 (86)	19 (14)	134	1		1	
	Partial	126 (81)	29 (19)	155	0.72 (0.38–1.35)	0.303	0.74 (0.35–1.60)	0.45
	None	21 (81)	5 (19)	26	0.70 (0.23–2.06)	0.511	0.43 (0.09–1.92)	0.27
Water source	Domestic	12 (63)	7 (36)	19	1			
	Rainwater	250 (84)	46 (16)	296	3.17 (1.19–8.45)	0.02	1.11 (0.28–4.39)	0.87

*Note*: Category used as reference (R = 1), social and environmental factors were dropped in the analysis they had less impact

*Abbreviations*: OR, odds ratio; CI, confidence interval, n, number of breeding sites

### Spatial and seasonal distribution of *Ae. aegypti* immatures

**Fig. 4:**
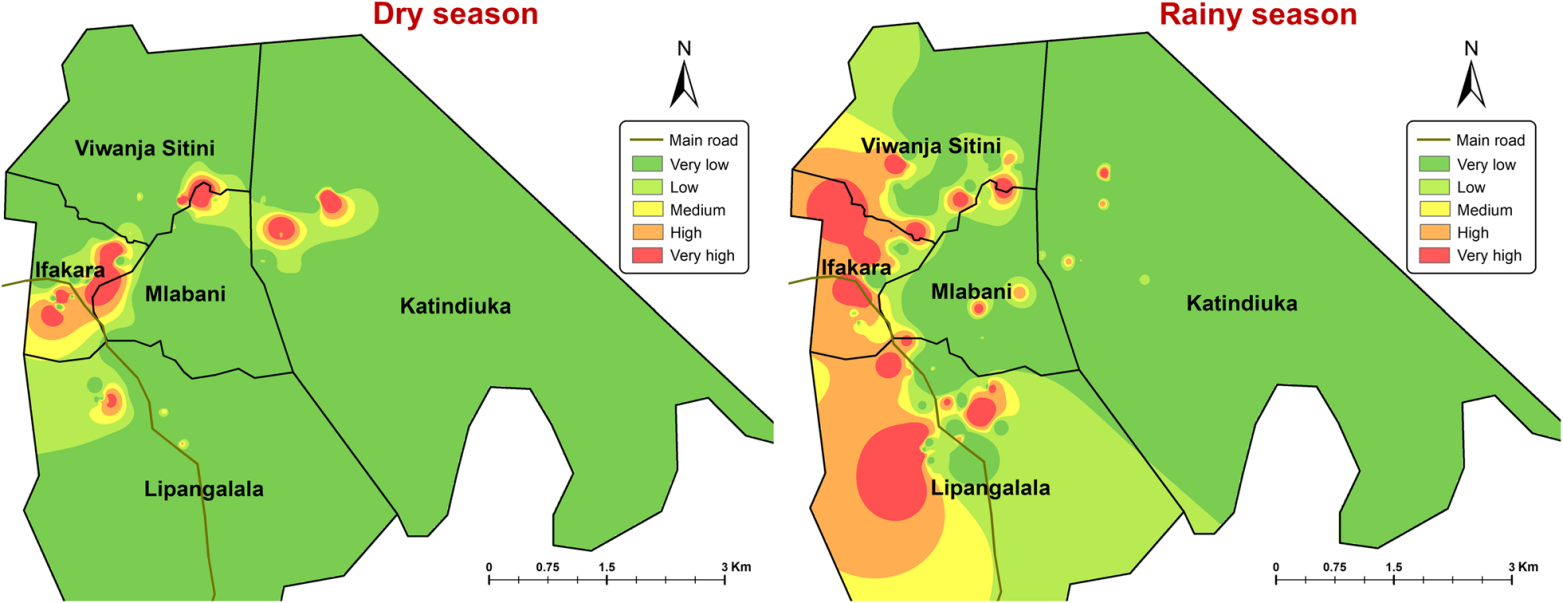
Spatial and seasonal distribution of *Aedes* larvae infested locations. *Key*: Very low (0–16 larvae/dip); Low (17–20 larvae/dip); Medium (21–23 larvae/dip); High (24–28 larvae/dip); Very high (29-37 larvae/dip)

The spatial distribution of *Ae. aegypti* immatures varied between the dry and rainy season (Fig. 4). In the dry season, the highest infestation was from the center of Ifakara town toward western parts of Katindiuka ward. In contrast, in the rainy season most infested locations were in southern Lipangalala and in Viwanja sitini (Fig. 4).

**Fig. 5:**
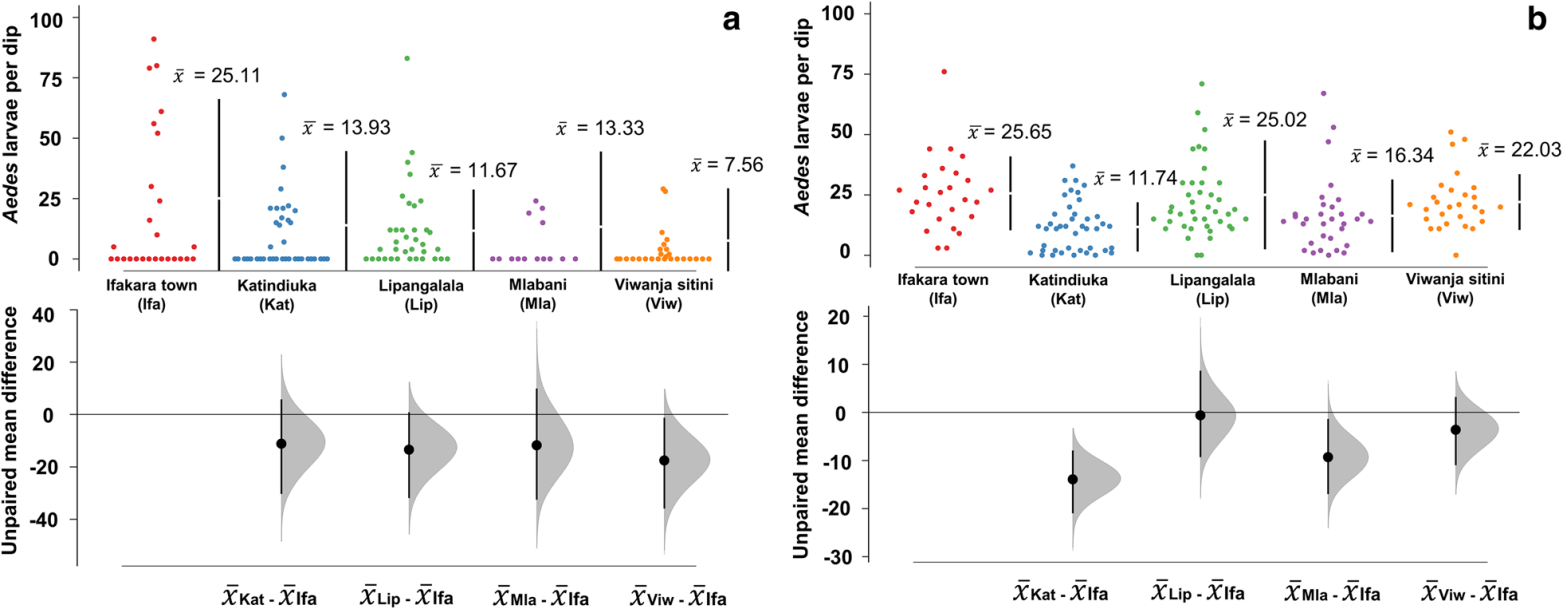
Estimated means of *Aedes* larvae/dip in Ifakara town and surrounding wards in the dry season (**a**) and the rainy season (**b**). Estimation plots are used to portray the distribution of residual mean differences of larval abundance between study wards. The vertical lines represent the mean ± confidence levels (the gap in the line is the mean). The filled curves indicate the resampled mean difference distribution of the larval abundances with reference to Ifakara town. Black vertical lines indicate 95% confidence level. Black dots indicate mean differences from the reference group. The significance is considered depending on how far the means of residuals deviated from the reference line

Generally, fewer breeding sites were observed in the dry season compared to the rainy season in all study sites, although the actual abundance varied significantly between sites. Ifakara town consistently had higher mean number of *Aedes* larvae than other wards across seasons (Fig. 5). The residual mean differences of larval abundance were estimated between study ward.

### Susceptibility of adult *Aedes aegypti* mosquitoes to insecticides

**Fig. 6:**
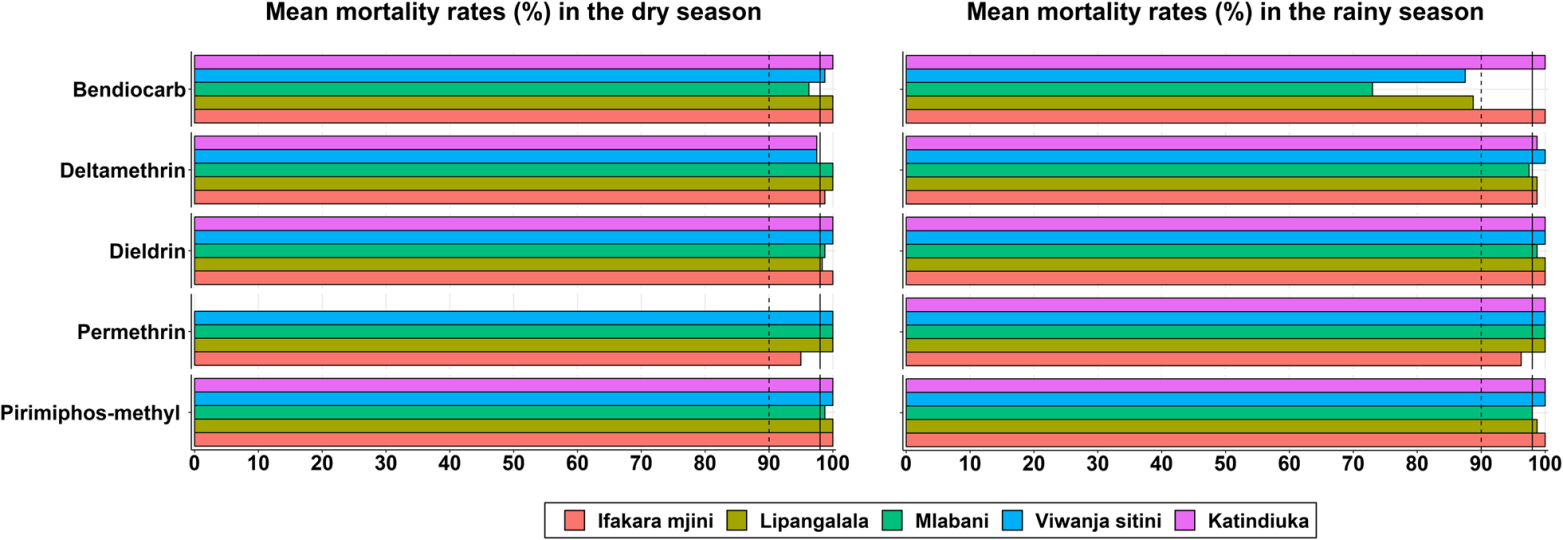
Mean mortality demonstrating susceptibility status of *Ae. aegypti* in the dry and rainy seasons. The solid lines (≥ 98% mortality) indicate that mosquitoes are fully susceptible to insecticide, while the dotted lines (90–98% mortality) indicate possible resistance requiring confirmation

*Aedes aegypti* females were generally susceptible to all four classes of insecticides. Only in a few instances did *Ae. aegypti* show reduced susceptibility to carbamates and pyrethroids (Fig. 6). Confirmed resistance was detected against only bendiocarb, and only in the rainy season (Fig. 6).

Overall knockdown KDT_50_ and KDT_95_ ranged from 7 to 112 min and 13 to 159 min respectively (Table 5). The knockdown analysis revealed spatial and seasonal variation. Dieldrin and pirimiphos-methyl consistently achieved slower knockdown across wards, while bendiocarb and deltamethrin exhibited quick knockdown. Knockdown times were not predictive of overall 24 h mortality. Often mosquitoes were not affected by the insecticides during first 60 min but mortality was still high after 24 h.

**Table 5:** Knock-down times of *Ae. aegypti* mosquitoes collected from different sites

Insecticide	Ward	Dry season		Rain season	
		KDT_50_ ± SE (min)	KDT_95_ ± SE (min)	KDT_50_ ± SE (min)	KDT_95_ ± SE (min)
Bendiocarb	Ifakara town	21.44 ± 4.52	28.68 ± 8.95	14.58 ± 6.28	30.26 ± 12.90
	Katindiuka	16.89 ± 3.05	22.15 ± 6.17	22.85 ± 6.68	39.34 ± 13.45
	Lipangalala	30.00 ± 5.82	41.16 ± 10.29	32.94 ± 8.04	53.86 ± 15.43
	Mlabani	25.13 ± 6.94	42.28 ± 13.66	30.77 ± 6.48	44.18 ± 11.70
	Viwanja sitini	28.91 ± 5.67	38.99 ± 10.08	39.77 ± 9.18	63.91 ± 18.76
Deltamethrin	Ifakara town	9.67 ± 3.56	14.11 ± 5.78	6.19 ± 5.9	17.16 ± 8.83
	Katindiuka	11.45 ± 4.42	19.95 ± 7.65	12.00 ± 9.60	37.44 ± 18.07
	Lipangalala	29.09 ± 46.16	31.59 ± 76.50	12.46 ± 3.44	18.52 ± 6.27
	Mlabani	7.20 ± 4.58	12.30 ± 5.27	16.41 ± 13.99	59.19 ± 30.42
	Viwanja sitini	7.12 ± 4.48	13.08 ± 5.75	17.64 ± 5.45	30.20 ± 11.55
Dieldrin	Ifakara town	36.02 ± 7.32	52.69 ± 13.26	75.43 ± 49.31	101.68 ± 103.19
	Katindiuka	40.73 ± 7.56	57.80 ± 13.96	22.90 ± 8.44	47.57 ± 17.40
	Lipangalala	43.32 ± 5.95	53.86 ± 10.68	85.57 ± 70.37	146.57 ± 154.15
	Mlabani	70.90 ± 33.93	102.46 ± 75.05	40.21 ± 8.70	62.23 ± 17.21
	Viwanja sitini	49.01 ± 6.89	62.59 ± 13.51	66.17 ± 370.89	70.70 ± 620.79
Permethrin	Ifakara town	12.69 ± 7.55	32.13 ± 14.93	7.20 ± 7.59	23.42 ± 12.58
	Katindiuka	–	–	10.56 ± 8.14	30.66 ± 15.25
	Lipangalala	8.52 ± 4.38	14.87 ± 5.95	12.28 ± 2.60	16.27 ± 4.68
	Mlabani	29.83 ± 7.21	47.19 ± 13.58	9.54 ± 8.73	30.60 ± 15.78
	Viwanja sitini	15.38 ± 4.41	24.73 ± 9.57	18.28 ± 3.17	23.45 ± 7.01
Pirimiphos-methyl	Ifakara town	75.66 ± 44.78	109.97 ± 95.41	71.03 ± 37.01	114.39 ± 83.36
	Katindiuka	78.03 ± 50.32	125.04 ± 109	26.66 ± 7.90	48.30 ± 15.75
	Lipangalala	79.14 ± 52.26	123.36 ± 111.14	32.36 ± 10.12	63.19 ± 22.59
	Mlabani	60.00 ± 15.83	84.06 ± 38.22	43.72 ± 8.41	63.61 ± 16.69
	Viwanja sitini	83.29 ± 102.4	108.97 ± 193.88	39.75 ± 14.95	84.60 ± 41.95

*Note*: In each experiment there were six replicates and 120–150 *Ae. aegypti* female mosquitoes

*Abbreviations*: SE, standard error; KDT_50_, time taken for 50% of the tested mosquitoes to be knocked-down; KDT_95_ time taken for 95% of the tested mosquitoes to be knocked-dow

### Wing length of adult *Ae. aegypti* mosquitoes

**Fig. 7:**
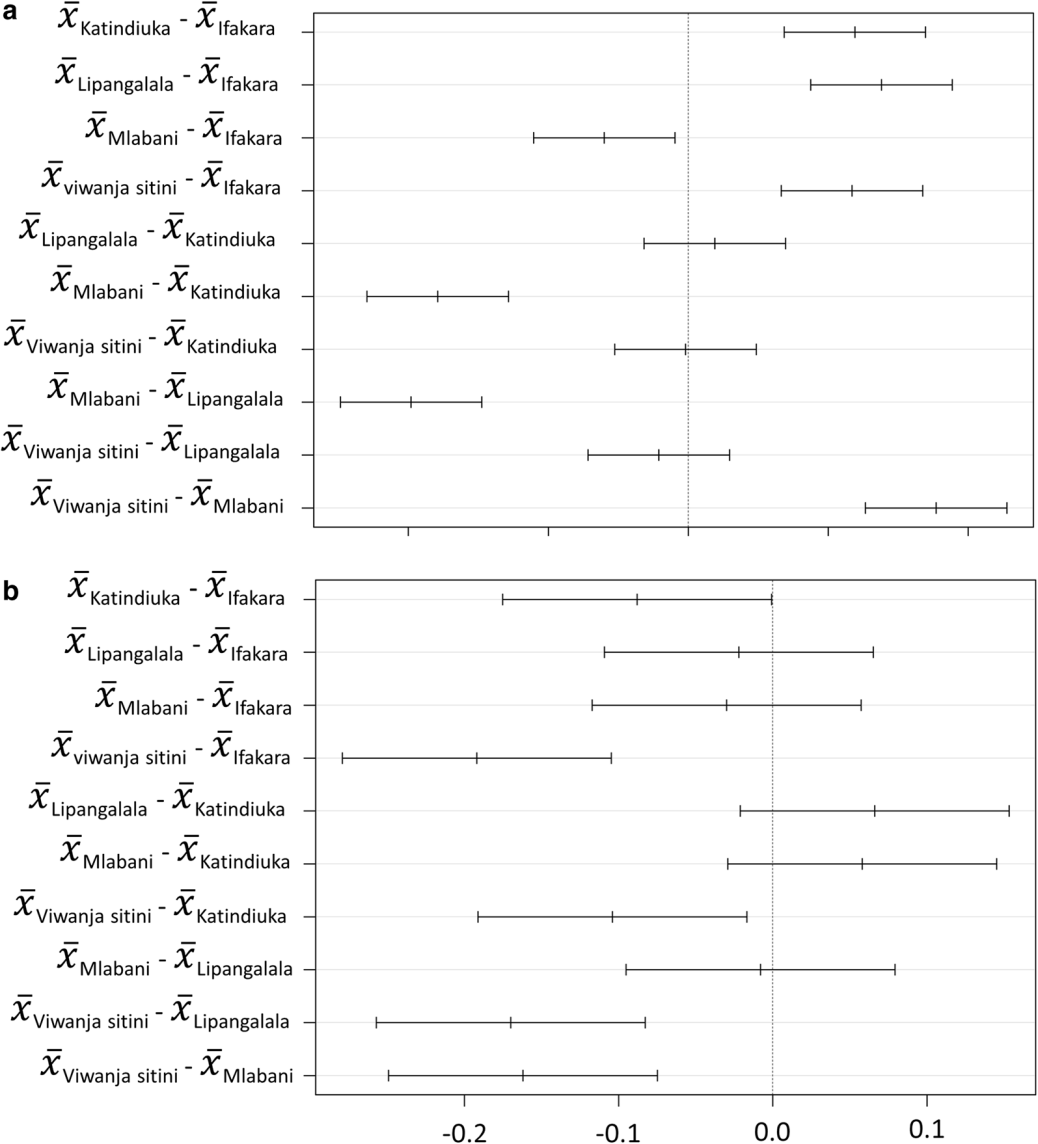
Differences in mean wing length between wards. Pairwise comparisons are shown at 95% confidence levels for female (**a**) and male (**b**) *Aedes aegypti* mosquitoes

Wing length, used here as a proxy for adult size of male and female *Ae. aegypti* ranged from 1.9 mm to 3.5 mm (Fig. 7). The mean (± SD) wing length was 2.48 ± 0.15 for mosquitoes from Ifakara town, 2.68 ± 0.23 in Katindiuka, 2.73 ± 0.20 in Lipangalala, 2.33 ± 0.18) in Mlabani and 2.68 ± 0.13 in Viwanja sitini. There was a significant difference in female mosquito wing length across wards (ANOVA: *F*_(4, 245)_ = 45.5, *P* < 0.001). *Post-hoc* analysis also revealed differences between pairs of wards (Fig. 7). Also, the mean wing length of female *Ae. aegypti* was generally larger than that of male *Ae. aegypti* (ANOVA: *F*_(1, 498)_ = 365.9, *P* < 0.001).

## Discussion

In Tanzania, the majority of studies conducted on arbovirus vectors are in response to outbreaks, and are often concentrated in large urban areas [11]. Basic ecological studies to understand the distribution and behavior of the vectors or their responses to interventions remain very few. This study involved an exploratory survey of *Ae. aegypti* mosquitoes in the small town of Ifakara and its surrounding wards in south-eastern Tanzania. The findings therefore constitute essential baseline data on *Ae. aegypti* mosquitoes in this area where no outbreak has previously been reported, yet the risk is high. Given previous reports of arboviral infections such as dengue and chikungunya in neighboring districts [7], it is crucial to invest on basic studies to improve our understanding of the ecology of the vectors, so as to boost control options.

The main finding was that larval indices, CI, HI and BI, are high enough to signal significant risk of *Aedes*-borne diseases in the area. In the rainy season in particular, house and container indices in all wards exceeded the value of 5.0, specified by WHO for actionable arboviral infections risk [38, 39]. Dry season risk was however confined to fewer wards though not completely absent from the rest of the wards. Immature *Ae. aegypti* infestation varied between wards and seasons, but remained significant even in the dry season. This was expected since *Aedes* mosquitoes typically breed in man-made containers not fully dependent on rainfall. Besides, the vectors have fewer options of breeding sites in the dry season hence elevating container level of infestation with immature *Ae. aegypti* (Table 1). On the contrary, aquatic habitats were relatively large in number during the rainy season, resulting in lower positivity rates (Table 1). This higher level of container infestation in the dry season concur with the study conducted in northern regions of Ghana which showed that, indices in the dry season were aggravated by poor water supply system in the area. As a result, water stored for domestic purposes stayed long enough to enable *Aedes* mosquito breeding [40].

Similar to earlier research, this study has shown that *Ae. aegypti* prefers breeding in clean and stagnant waters [19, 20, 41]. Common habitats for *Ae. aegypti* were used tires, clay pots, flowerpots, containers, coconut tree-holes, pits, and on rare occasions disposed shoes, cooking pans, broken grasses and tarpaulins. The majority of these habitats were easy to discard, indicating an opportunity for proper waste management and environmental management as effective options for *Aedes* control, especially if used alongside traditional larviciding. As already highlighted by several previous studies, tires in particular serve as important breeding sites for *Ae. aegypti* because they can hold water for long periods even in the dry season [11, 19, 42]. The multiple applications of used tires in the area will, however, complicate efforts to effectively dispose of the tires. For example, people use these tires as make-shift chairs, for playing by kids, for planting trees (residents believed that tires prevent plant pests), and for vehicle repairs.

A major natural breeding site in the area was coconut trees, which had artificial holes created for climbing during the coconut harvesting period. These holes served as the perfect breeding sites for *Ae. aegypti* mosquitoes. This study therefore recommends that coconut tree-holes be filled with sand to prevent rainwater from stagnating. Clay pots were also common in Katindiuka and Lipangalala wards, where they were mostly used for collecting rainwater for domestic use. Unfortunately, residents were not adequately aware that these pots are breeding sites for *Ae. aegypti* mosquitoes. Rare habitats such as disposed coconut shells, broken glass, animal feeding containers, tarpaulins and discarded plastic shoes were also observed, which produced high larval abundance (larvae/dipper). Larval abundance was influenced by the size of habitats and the volume of water present in breeding sites.

During the data collection period, surrounding communities were explained about mosquito breeding behaviors and diseases they transmit to raise awareness. This led to a better understanding and greater engagement of the communities in this work. Some breeding sites observed during the first visit were not there during subsequent visit as people became aware of the risks and hence proactively removed or covered potential habitats. This observation highlights the potential of educating communities about breeding sources of *Ae. aegypti* and participatory control efforts. In Tanzania, the government is already implementing monthly clean-up campaigns, which could be leveraged to achieve such gains. Moreover, efforts to reduce mosquito population can prioritize areas identified with higher risk.

Mosquito sizes play an important role in overall ability of the individual mosquitoes to pick up and disseminate pathogens, including viruses [43, 44]. For example, smaller mosquitoes tend to have more contacts with hosts as they need more frequent blood meals than larger mosquitoes, a phenomenon which could increase transmission [44]. In the present study, the wing length measurements for *Ae. aegypti* ranged between 1.9–3.5 mm. The differences between administrative wards may imply that some of the wards could harbor vectors with greater vectorial capacity than others, but the actual extent to which such variations affect pathogen spread in this area remains to be investigated. On the other hand, larger mosquitoes have been demonstrated to be more resistant toward insecticides [45], which may also influence susceptibility of those mosquitoes to public health insecticides. This study therefore provides a baseline assessment of wing lengths, so that any future changes can be compared against current status of the insecticide-susceptible *Ae. aegypti* populations. It is recognized that statistical variability in wing length measurements may be greater among mosquitoes collected at their aquatic stages compared to those collected as adults [46]. However, in this study all wing measurements were taken on mosquitoes collected at aquatic stages, thus minimizing any potential differences associated with the mosquito life-cycle stage.

Finally, *Ae. aegypti* mosquitoes in the area were assessed for how they would respond to control by commonly available insecticides. Fortunately, this study showed that the mosquito populations in the Ifakara area are still generally susceptible to most insecticide classes. Given that *Ae. aegypti* mosquitoes are mostly outdoor bitters [47], their exposure to chemical indoor interventions such as insecticide-treated nets is likely low. Interestingly, it was observed that in a few wards, the mosquitoes were resistant to bendiocarb during the rainy season but not in the dry season. Future research should examine potential environmental drivers and the extent of these phenotypes. Previous research has already demonstrated fine-scale spatial and temporal differences in insecticide susceptibility of both *Anopheles* [48] and *Culex* mosquitoes [49] to pesticides.

Since this study was the first of its kind in this geographical area, there were no immediate comparisons for the resistance profiles. However, in studies carried out in Dar es Salaam, Peru and Burkina Faso, resistance to pyrethroids and organophosphate was obvious [20, 50, 51]. Reduced susceptibility to pyrethroids observed in some of assays, and the resistance seen against bendiocarb in the rainy season are however signs that we must remain vigilant as insecticide resistance could rapidly spread among the vector populations once active control programmes begin. This would therefore mean that environmental management, including larval habitats search and removal, should be an important component of any anti-*Aedes* campaigns. As most habitats are those that can be discarded, combinations of insecticidal and non-insecticidal approaches would likely be effective.

Although the main objectives were successfully completed, this study also had various limitations. First, larvae and pupae were only collected in the selected grids, yet the wards were not of the same surface area (Fig. 2). It is possible therefore that some of the *Aedes* indices might have been underestimated. This study therefore recommends that future studies should consider all the grids occupied by human habitations and building.

Secondly, the WHO standard dose specified for *Anopheles* mosquitoes was adopted in this study, as there is still no comprehensive WHO guideline for assessing susceptibility of *Aedes* mosquitoes. However, some of these insecticides, such as pirimiphos-methyl, permethrin and deltamethrin already have diagnostic concentrations specific for *Aedes* mosquitoes. Therefore, if the right concentration had been used, the results might have been different. For instance, results for permethrin (0.75%) demonstrated susceptibility toward standard concentration for *Anopheles*, which is three times the *Aedes* standard concentration (0.25%) indicating that the result of permethrin obtained here might have been overrated. This study therefore recommends that future studies should incorporate appropriate guidelines for the species.

## Conclusions

To our knowledge, this is the first study on ecology and insecticide susceptibility of *Ae. aegypti* in the Ifakara area, and will provide a basis for future evaluation of its role in pathogen transmission, as well as options for its control. Infestation levels observed indicate that immediate vector control campaigns, coupled with targeted surveys of *Aedes*-borne diseases in humans, should be conducted to track and prevent potential outbreaks. The larval indices (container index, house index and Breteaux index) are high enough to signal significant risk of *Aedes*-borne diseases in the area. Fortunately, *Ae. aegypti* in the area are still susceptible to majority of insecticides used in public health, indicating there are still opportunities to include insecticides in the *Aedes* control programmes. Since most habitats were those that can be discarded, integrating concepts of environmental management, insecticide use, and community engagement could yield significant progress. While used tires, discarded containers and flowerpots are key habitats for *Aedes* in the area, this study also identified coconut harvesting as an important risk factor, and the associated tree-holes as vital targets for *Aedes* control.

## Data Availability

Data supporting the conclusions of this article are included within the article. All raw data for this study will be available upon request the lead author (NFK).

## Abbreviations

ANOVA: analysis of variance
BI: Breteaux Index
CI: Container Index
CIESIN: Centre for International Earth Science Information Network
ESRI, USA: Environmental Systems Research Institute, USA
GPS: geographical position system
GVCR: Global Vector Control Response
HI: House Index
HRSL: high resolution settlement layer
IDW: inverse distance weighted
IHI: Ifakara Health Institute
KDT: knock-down time
MRCC: Medical Research Coordinating Committee
NIMR: National Institutes of Medical Research
OR: odds ratio
RR: relative risk
WHO: World Health Organization
